# A Preliminary Assessment of Size-Fractionated Microplastics in Indoor Aerosol—Kuwait’s Baseline

**DOI:** 10.3390/toxics10020071

**Published:** 2022-02-04

**Authors:** Saif Uddin, Scott W. Fowler, Nazima Habibi, Sufiya Sajid, Sam Dupont, Montaha Behbehani

**Affiliations:** 1 Environment and Life Sciences Research Center, Kuwait Institute for Scientific Research, P.O. Box. 24885, Safat 13109, Kuwait; nhabibi@kisr.edu.kw (N.H.); ssajid@kisr.edu.kw (S.S.); mbahbaha@kisr.edu.kw (M.B.); 2 School of Marine and Atmospheric Sciences, Stony Brook University, Stony Brook, NY 11794-5000, USA; fowlerscottw@yahoo.com; 3 Institute Bobby, 8 Allée des Orangers, 06320 Cap d’Ail, France; 4 Department of Biological and Environmental Sciences, University of Gothenburg, 405 30 Gothenburg, Sweden; sam.dupont@bioenv.gu.se; 5 Environmental Laboratories, International Atomic Energy Agency, 4 Quai Antoine Ier, 98000 Monaco, Monaco

**Keywords:** airborne microplastic, active sampling, passive sampling, indoor, outdoor, aerosol, atmospheric fallout, dust

## Abstract

The omnipresence of microplastic (MP) in various environmental samples, including aerosols, has raised public health concerns; however, there is presently very limited information on MPs in indoor aerosol. This paper presents a unique dataset where smaller MPs have been sampled using a six-stage cascade impactor from indoor environments in Kuwait. The MP concentration in the indoor air varied between 3.2 and 27.1 particles m^−3^, and the relative MP concentration decreased linearly from the lowest to the highest size fraction. A significant effect of location was observed for the total number of MPs (F_2,14_ = 5.80, *p* = 0.02) and the inhalable fraction (F_2,14_ = 8.38, *p* = 0.005), while location had no effect on the respirable fraction (F_2,14_ = 0.54, *p* = 0.60). A significant effect of the type of air conditioning used was also observed for the total number of MPs (F_2,19_ = 5.58, *p* = 0.01) and the inhalable fraction (F_2,19_ = 6.45, *p* = 0.008), while location had no effect on the respirable fraction (F_2,19_ = 1.30, *p* = 0.30). For the total number of MPs and the inhalable fraction, the concentration was significantly higher for the split unit air-conditioning as compared to the central air-conditioning plants. The presence/absence of carpets had no significant effect on the MP concentrations (total: F_1,19_ = 4.08, *p* = 0.06; inhalable: F_1,19_ = 3.03, *p* = 0.10; respirable: F_1,19_ = 4.27, *p* = 0.05). The shape was dominantly fibers, with few fragments in lower size fractions. These datasets represent the first baseline information for Kuwait, and the smaller MPs in all the samples further underscore the need to develop standardized protocols of MP collection in the ≤2.5 µm fraction that can have more conspicuous health implications.

## 1. Introduction

The persistent nature and omnipresence of microplastic (MP) in the aquatic environment has attracted massive attention from the scientific community. Several thousand publications on MP in the aquatic environment have been published since 2004, when the term was first introduced. In contrast, research on MP in aerosols remains less explored, with less than two dozen studies on outdoor air [1,2,3,4,5,6,7,8,9,10,11,12,13,14,15,16,17,18,19,20,21,22] and only a few on indoor air [1,14,23,24,25,26,27,28].

The presence of microplastics in the air has been related to release from clothing, furnishings, synthetic tires, and degraded plastics, among other causes [1,2,3,6,17,29,30,31,32,33,34]. Aerosols can be a significant pathway for transferring MPs to humans via inhalation [4,23,35,36,37,38,39]. The ecological concern from microplastics emanates from the fact that they can be inhaled by humans and can potentially lead to adverse health effects, such as localized inflammation [40], genotoxicity [4], and the development of oxidative stress and cytotoxicity [36]. For this reason, it is prudent to look at the finer MPs that can be inhaled [41]. Considering the lower size cutoff for microplastics i.e., 1 µm [42], the fine MPs have greater potential to be transferred via aerosolization into the human respiratory system [17].

A few studies on airborne microplastics gained more attention when they suggested human health risks due to MP inhalation, most importantly, the respirable (PM_10_) and inhalable (PM_2.5_) fractions that can reach deep in the lungs and may be taken up by both macrophages and epithelial cells [43,44]. Some studies reported that fibrous MPs up to 250 µm in size avert the lung’s clearance mechanisms [45]. Several health issues were reported, including reduced lung capacity in work-related conditions, coughing, and breathlessness [46,47,48]. Two recent reviews have highlighted the potential effects on human health [38,39].

A study reported that microplastics deposition is more likely to occur in the upper airway tract (i.e., nose, mouth, throat) and can reach the gut when swallowed [31]. Inhaled or ingested finer MPs are believed to be able to translocate to the circulatory system and other organs [40]. The plastic additives, dyes, and pigments could lead to reproductive toxicity, carcinogenicity, and mutagenicity [49,50]. Over 4000 chemicals are currently used in the plastic food packaging industry itself [51], and can provide a scale of the chemical toxicity they can induce. Most of the additives added to the plastic during processing are of small molecular size and often not chemically bound to the polymeric materials, which make them susceptible to leaching into the surrounding environment [36]. There is voluminous literature available on the sorption ability of MP [3,35,52,53,54,55,56,57,58,59,60,61,62,63,64,65,66,67,68,69,70]. It has been reported that polycyclic aromatic hydrocarbons (PAHs) and metals released from traffic emissions, as well as microorganisms, adhere to MP surfaces and may be transferred directly to the human lung [4].

It has been estimated that about 89% of modern human activities are conducted in the indoor environment [71]; hence, it is prudent to monitor indoor environments. Some studies have reported a higher indoor concentration of suspended and deposited MPs compared to the outdoors [1,13]. Unfortunately, there have only been seven studies conducted so far on MP in indoor aerosol; four of these studies have sampled indoor air using active samplers [1,23,27,28] and the other three collected indoor dust from non-carpeted apartments and the East China Normal University [14,24,25,26].

The present study is the first attempt to generate data on MP in Kuwait’s indoor aerosols, which addresses the larger issue of the paucity of MP data in indoor air and considers the fact that due to the extremely hot and arid climate in Kuwait, people spend most of their time indoors in an air-conditioned atmosphere where the fresh air input and exchange is very limited.

## 2. Sample Collection and Preparation

In this study, a six-stage ambient viable cascade impactor from Tisch Environmental, model TE-10-800, was used for sample collection. The air was drawn at 30 L min^−1^ for 360 min. The samples were collected directly onto the impactor plates without using any substrate and collected samples were microscopically identified and quantified with further verification using hot needle and micro-Raman spectroscopy.

The sample preparation of microplastic in aerosol samples is a critical step, since the aerosol samples include various inorganic (mineral grains) and organic matter (pollen, fungi, microbes, soot, etc.) that might be difficult to segregate. The removal of MP from the other particulate aerosols is a crucial step for accurate identification and spectrographic characterization [72]. However, in this study, we have used a slightly different approach of collecting samples directly on an aluminum alloy, pre-cleaned cascade impactor, without using any intervening substrate. This implies that the likely loss of material or leaching of particles from the collecting substrate was minimized. Once the samples were collected, each of the collected impactor plates was subjected to a microscopic examination, all the particles were meticulously collected using an electrostatically charged ultrafine glass rod and in some cases using forceps, and placed on a pre-cleaned glass slide. This was done in a lamellar flow cabinet. The blank and control samples were also processed along with collected samples.

## 3. Identification of Microplastics in Aerosol Samples

The microplastics on these slides were identified using a multi-tier process. An initial visual examination was carried out using a fluorescence stereomicroscope (Leica DM2500 LED) at 40/0.75X to 1000X magnification, sorting out particles based on the absence of cellular structure and thickness consistency along with their length, relatively homogenous coloring, and transparency. These particles were counted and their size was measured using ImageJ software, however, there was no measurement made for particles below 5 µm, which were transferred directly to another glass slide using an electrostatic charge to be observed under scanning electron microscope later.

The second tier of identification was done after these particles were strained with Nile red and MP identification was done under a UV stereomicroscope using a hot needle technique [73,74]. The hot needle test works effectively as the plastic pieces curl and deform on touching, while the other non-plastic materials will not. About 50% of the samples were subjected to the hot needle test. As part of the quality assurance procedure, a blank sample and a positive control were also examined with each collection.

We also attempted polymeric characterization of the MPs on ~10% of samples selected randomly. The sample fraction used for polymeric characterization was taken from stages 1–5, and micro-Raman spectroscopy was used [15]. Micro-Raman was preferred as it can be used to identify microplastics up to 1 μm in size [16] and smaller MPs are more relevant for human health assessments. However, we have not reported the polymeric data in this communication as we believe it requires further processing to remove concurrent fluorescence interference that we believe is due to coloring agents. The spectra observed using 785 nm excitation laser showed that most of the larger fibers were polyester and nylon, and fragments were acrylic and polyurethane.

## 4. Microplastic in Indoor Air

An initial assessment of the microplastic in indoor aerosol in Kuwait was done by sampling several different types of sites, including public/government buildings, residential dwellings of different types spread over the city, a hospital, and a mosque. Sampling was carried out over an approximately 9 month period from January to the beginning of October, 2021. Air was drawn through a six-stage cascade and the orifice dimensions and size fractions for each stage are given in Table 1. The aerosol was drawn at 30 L min^−1^ for 360 min, resulting in collection of 10.8 m^3^ of aerosol. Table 2 presents the number of MPs in size-fractionated aerosols and their concentrations per m^3^ of aerosol. The focus of this study was to establish a baseline on size-fractionated MPs in aerosol, since most of the human health assessments are based on the size fractions rather than chemical composition.

The MP concentration in the indoor air in Kuwait varied between 3.2 and 27.1 particles m^−3^. The government/public buildings had a concentration of 3.2–15.3 MP m^−3^, the lower concentrations of 3.2 and 3.7 MP m^−3^ were during the time of excessive restrictions, when no more than 100 employee/visitors were permitted. In the same buildings, the concentration of MPs in indoor aerosols increased from 8.7–15.3 MP m^−3^ when the staff and visitor number increased to 300–1000. Higher concentrations, between 8.2–11.0 MP m^−3^, were also observed in carpeted offices.

The MP concentrations in the indoor aerosol samples of residential dwellings were much higher; the carpeted flats had MP concentrations of 10.8–27.1 MP m^−3^, while in houses with low occupancy (four persons) it was 6.3–13.0 MP m^−3^. The mosques in Kuwait are centrally air-conditioned with a thick carpeted floor and due to COVID-19 restrictions, everyone was required to bring their own prayer mat. The MP concentration in the mosque was 14.3 MP m^−3^. The MP concentration in hospital air was also observed to range between 3.9–4.4 MP m^−3^. We believe the use of PPEs, including disposable coveralls, facemasks, and hospital sheets are also contributing to the MP load in hospitals.

The size of MPs observed and measured under microscope were found to vary from 0.45 µm to 2800 µm. The size fractionation showed that the most dominant size class by enumeration was in the two size fractions >7 and 4.7–7.0 µm. These are quite different from other studies, because none of the other studies have used a cascade impactor with these cut-off sizes. The presence of ultrafine MPs is certainly a matter of huge concern from the human health perspective, more specifically for people in Kuwait who spend most of their time indoors throughout the year, but also for other countries where the indoor–outdoor air exchange is limited, especially during the winter months.

**Figure 1:**
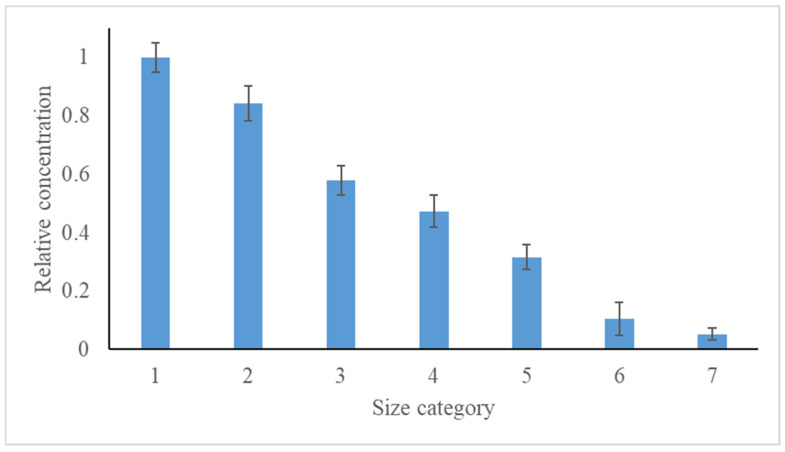
Relative concentration of MP in each size category.

Statistical analyses were performed using SAS. Differences between categories (location, presence of carpet, and type of air conditioning) were tested using an ANOVA model, followed by Scheffe’s post hoc tests. All data are presented as mean ± standard error of mean. The relative MP number concentration decreased linearly from the lowest to the highest size fraction (Figure 1).

**Figure 2:**
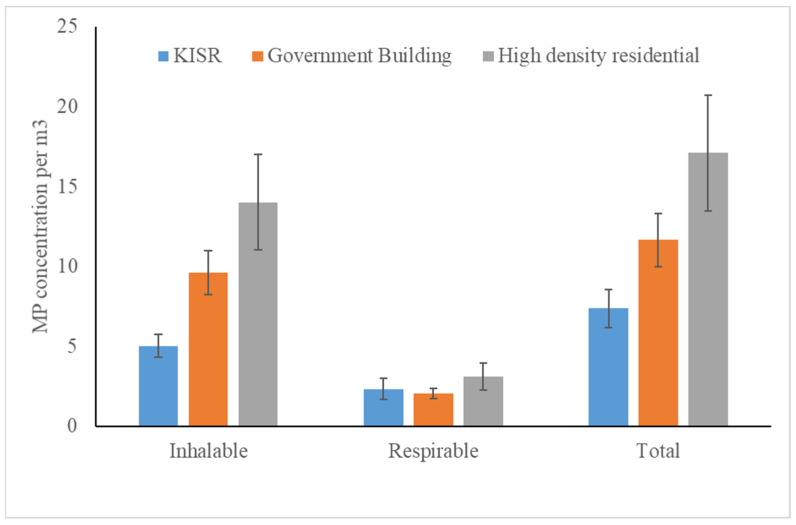
MP concentrations at three different locations (KISR, government building, and high-density residential building) for the inhalable, respirable, and total size fractions.

A significant effect of location was observed for the total number of MP (F_2,14_ = 5.80, *p* = 0.02) and the inhalable fraction (F_2,14_ = 8.38, *p* = 0.005), while location had no effect on the respirable fraction (F_2,14_ = 0.54, *p* = 0.60). When significant, the MP concentration was lower at Kuwait Institute for Scientific Research (KISR) as compared to the high-density residence (Figure 2).

**Figure 3:**
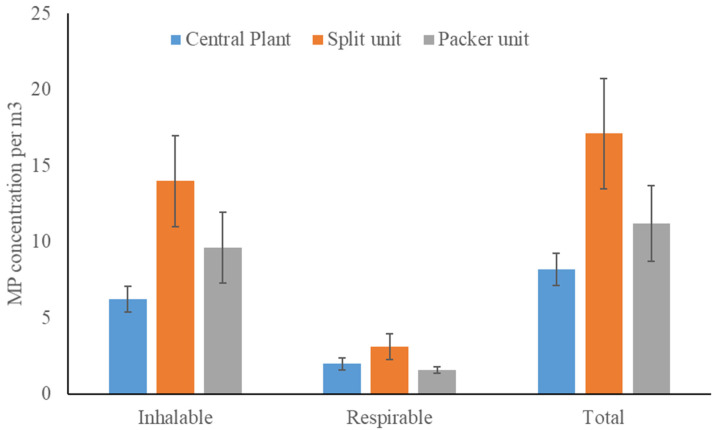
MP concentrations for three types of air conditioning (central plant, split unit, and packer unit) for the inhalable, respirable, and total size fractions.

A significant effect of the type of air conditioning was also observed for the total number of MPs (F_2,19_ = 5.58, *p* = 0.01) and the inhalable fraction (F_2,19_ = 6.45, *p* = 0.008), while location had no effect on the respirable fraction (F_2,19_ = 1.30, *p* = 0.30). For the total number of MPs and the inhalable fraction, the concentration was significantly higher for the split unit as compared to the central plant (Figure 3).

**Figure 4:**
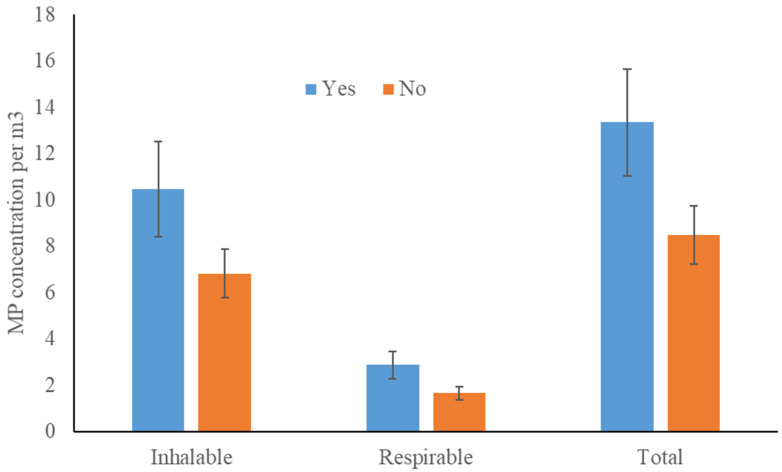
MP concentrations in presence/absence of carpet (yes/no) for the inhalable, respirable, and total size fractions.

The presence/absence of carpet had no significant effect on the MP concentrations (total: F_1,19_ = 4.08, *p* = 0.06; inhalable: F_1,19_ = 3.03, *p* = 0.10; respirable: F_1,19_ = 4.27, *p* = 0.05; Figure 4).

**Figure 5:**
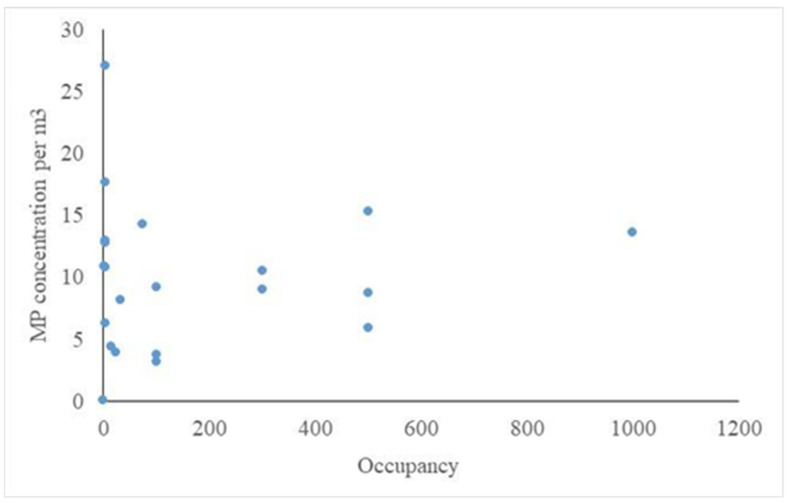
Relationship between the occupancy (number of people present) and the MP concentration.

No significant linear relationships were observed between the occupancy and the MP concentration (total: F_1,20_ = 0.23, *p* = 0.64; inhalable: F_1,20_ = 0.08, *p* = 0.78; respirable: F_1,20_ = 0.11, *p* = 0.74; Figure 5).

Studies have reported a higher indoor concentration of suspended and deposited MPs compared to outdoors [1,13]. A limited number of studies have been conducted for assessing MPs in indoor environments, however a direct comparison between these studies will not be meaningful as they have used different sampling strategies and sample processing techniques (Table 3).

The shape, size, color, and polymer type of MPs in the indoor environment have been reported by most of the prior studies. The concentration of MPs in the indoor samples have been highly variable; 0.4–59.4 MP m^−3^ was reported from Paris, France, whereas 1583 ± 1181 MP m^−3^ were reported from Wenzhou, China, with the most dominant size range of 50 and 200 µm.

Diverse shapes, including fiber, foam, fragments, and film, have been detected in the atmospheric microplastics, with fibers being the dominant shape (Table 4). Fiber was the most dominant shape in indoor samples in France, 39 cities in China, Australia, Portugal, and in this study. However, in another study from Wenzhou, China, the indoor aerosol had ~80% fragments and 10% fibers, similar to the situation in Aarhus, Denmark, where MPs in indoor aerosols were predominantly fragments (87%), with fibers constituting only 13%. In Hamburg, more than 90% of MPs detected were fragments and less than 10% were fibers, (Klein and Fischer 2019). MPs in the air from Chinese research reported 67−80% fibers, <30% fragments and <3% granules. Details of the shapes are also provided in Table 4.

The microplastics consisted of different colors, with most dominant ones being red, orange, yellow, white, grey, blue, black, green, and transparent. The use of color to identify potential sources of plastic debris is sometimes practiced [42], however, this can be quite misleading. Several studies have reported the colors of the identified MPs and these are summarized in Table 4. Blue and red MPs were reported from Paris [2], while black, blue, red, transparent, brown, green, yellow, and grey particles were reported from Shanghai, China [11].

A study done in Paris reported much higher indoor concentrations, ranging from 1 to 60 fibers m^−3^, as opposed to significantly lower outdoor concentrations ranging between 0.3 and 1.5 fibers m^−3^ [1]. In any case, exposure to microplastics concentrations has been shown to be higher on average in indoor environments than outdoor ones due to the former incurring more sources that allow several factors (e.g., ventilation and airflow) to influence MPs’ behavior and elevate their levels [36,75]. By contrast, MP concentrations in the latter environment are subjected to dilution from outside air, and therefore, exposure to lower MP levels is expected [1]. In addition, people spend 70–90% of their time indoors, which enhances exposure levels. Interestingly, microplastics generated indoors can frequently contaminate the environment outdoors, whereas only 30% of particulate matter produced outdoors can penetrate the indoor environment [75]. This underscores the importance of the indoor environment as the main exposure source of airborne microplastics [36].

## 5. Conclusions and Future Perspectives

This study confirms the presence of microplastic in the indoor air-conditioned buildings across Kuwait, a hyper-arid country, where most of the activities are indoor. The study also adds to the limited data on MPs in indoor aerosols; however, the concentrations vary across different types of buildings, depending on type of air conditioning. This study also provides an insight into the MP distribution within the inhalable and respirable fractions of aerosols, considering the 50% cut-off size for aerodynamic size fractions of 2.5 µm, and it provides evidence for a much higher inhalable fraction, roughly a factor of three more than the respirable fraction. The need for <10 and ≤2.5 µm data for aerosols was highlighted as being a potentially important dataset for human health assessment [34].

The data from studies looking at MPs cannot be directly compared as each one used a very different approach. In spite of the un-harmonized methodologies employed within all these studies, some reasonable observations can still be inferred. It could be summarized that fibers and fragments are the predominant shapes of MPs in indoor aerosols, while transparent and black were the most prevalent colors. It is quite evident that due to lack of standardized methodologies, the atmospheric microplastics research certainly lacks sufficient comparable data. With the use of active and passive sampling strategies, the reporting units are very different and often cannot be compared. The passive sampling also provides insufficient information for inhalation risk assessment. Our study provides a first dataset on size-fractionated MPs in indoor aerosols.

Another important discussion we would like to bring up is that regarding the polymeric characterization of MPs—there have been many concerns raised on the methodologies followed and amount of information provided for assessing the data quality [77]. On the other hand, we would also like to question the relevance of the generated information regarding polymers, when the health risk assessments are not using the polymer type but the size, hence suggesting that just assuming that the particle detected is plastic is sufficient? [31].

Based on the experience gained in MP research we would like to highlight that some important points to consider for future work are:

(1) Active and passive sampling techniques should be used jointly for better assessment of both short-term and long-term atmospheric MP accumulation rates, respectively, particularly when the aspect of human health risk is investigated.

(2) Concentrations of 5–15% H_2_O_2_ or KOH ought to be utilized as opposed to 30% H_2_O_2_ for extruding organic matter from the collected samples in order to avoid the significant deterioration of polymers’ physical and chemical properties.

(3) A microscopic identification should be preferred instead of density separation using, for example, NaCl, NaI, etc. for particles >5 µm.

(4) The choice of filter is critical, and we recommend that it is better to use a cascade impactor without a filter.

(5) Polymer characterization should be taken up for 5–10% of the samples, depending on the size of MPs, one of the techniques, i.e., attenuated total reflectance Fourier transform infrared spectroscopy (ATR-FTIR), micro-Fourier transform infrared spectroscopy (µ-FTIR), and/or micro-Raman spectroscopy (µ-RAMAN) should be used. However, whether this information will be useful for human health risk assessments is not obvious.

(6) The microplastics in aerosol fractions should be reported for the total number of particles per unit volume of air (number of MP m^−3^) and the number of MPs in each of the aerodynamic classes. Such volumetric measurements will be more useful for human health assessments and the estimation of inhalation doses.

(7) There is a need to harmonize and standardize the methodology for sample collection, preparation, identification, and reporting of atmospheric microplastics. Moreover, research on the health risk implications to humans is an essential step that can be further accomplished by understanding the interactions between contaminants and microplastics, and their pathways of transfer and eventual exposure to humans.

## Figures and Tables

**Table 1:** Jet orifice dimensions and particle size range of the cascade impactor used for sampling.

Stage of Impactor	Orifice Diameter (mm)	Particle Size Range Captures (µm)
1	1.18	>7.0
2	0.91	4.7–7.0
3	0.71	3.3–4.7
4	0.53	2.1–3.3
5	0.34	1.1–2.1
6	0.25	0.65–1.1
Base plate 7	0.10	<0.65

**Table 2:** Number of microplastics in different size fractions in 10.8 m^3^ of aerosol.

Building Type	Date	Location	1	2	3	4	5	6	7	Total	MP m^−3^
Government/Public Buildings	6 January 2021	KISR Building No. 44 corridor (Laboratory 1501)	19	16	11	9	6	2	1	64	5.9
	5 April 2021	KISR main entrance * (~100 employees)	5	11	7	9	4	4	0	40	3.7
	21 April 2021	KISR Lobby * (~100 employees)	6	7	5	9	4	3	1	35	3.2
	4 May 2021	KISR corridor building No. 44 (eastside)	7	16	18	19	9	21	9	99	9.2
	14 March 2021	KISR office (Carpeted) Room 2205	12	14	16	19	26	31	0	119	11.0
	25 July 2021	KISR office (Carpeted) Room 2200	29	16	9	6	16	12	1	89	8.2
	17 May 2021	KISR main entrance (~300 employees)	20	27	19	20	19	8	0	113	10.5
	14 April 2021	Government building with about ~l000 visitors	25	39	38	21	15	8	1	147	13.6
	27 April 2021	Attendance section office building with ~500 employees	48	44	22	21	19	8	3	165	15.3
	6 July 2021	Reception of a government building ~500 visitors	16	18	26	21	6	4	3	94	8.7
	5 October 2021	Attendance section of office building ~300 employees	32	9	18	17	11	8	2	97	9.0
Private housing, low and high density	14 March 2021	High density residential (carpeted flat) Hawally	59	36	28	18	22	18	10	191	17.7
	14 June 2021	High density residential (carpeted flat) Abu Haleefa—9th floor	75	62	59	49	28	11	9	293	27.1
	15 June 2021	High density residential (carpeted flat) Jaleeb—8th floor	49	19	20	13	9	5	2	117	10.8
	19 June 2021	High density residential (carpeted flat) Regga—1st floor	39	41	24	14	8	7	5	138	12.8
	25 June 2021	House with low occupancy (rugs) Daiya Area	42	39	28	16	8	6	1	140	13.0
	3 August 2021	House with low occupancy (rugs) Shuwaikh Area	20	11	13	10	8	5	1	68	6.3
Hospital	8 January 2021	Causality ward Sheikh Jaber Hospital	12	14	9	4	1	2	0	42	3.9
	29 June 2021	COVID ward of Sheikh Jaber Hospital	10	9	11	7	6	4	0	47	4.4
Mosque	1 September2021	Mosque carpeted Eagila	45	39	32	16	11	7	4	154	14.3
		Control (used for identification only)	5	5	5	10	10	10	10	55	
		Blank 1 (exposed to indoor air—passive)	1	0	0	0	0	0	0	1	
		Blank 2 (exposed to indoor air—passive)	2	0	0	0	0	0	0	2	
		Blank 3 (exposed—passive)	0	0	0	0	0	0	0	0	
		Blank 4 (exposed—passive)	1	0	0	0	0	0	0	1	

KISR = Kuwait Institute for Scientific Research, * during COVID-19 partial lockdown.

**Table 3:** A summary of sampling techniques and processing techniques used for the extraction of microplastic from the aerosol samples.

City/Country	Sample Matrix	Sampling Technique	Sampling Extraction/Treatment	Reference
Kuwait	Aerosol	Active Sampling: Sampling using 6-stage compactor, sample drawn at a rate of 30 L min^−1^. A total of 10.8 m^3^ of aerosol was drawn.		This Study
Paris, France	Aerosol	Active Sampling: A pump for drawing indoor air (8 L/min) and quartz fiber GF/A Whatman filters (1.6 mm, 47 mm) for sample collection (2–5 m^3^) from two apartments and an office.	Samples passed through a 2.5 mm mesh size sieve and the retained fraction (>2.5 mm) visually inspected to verify plastics presence. Mass of 5.5 mg introduced in a separation funnel with 50 mL of ZnCl_2_ for density separation. Floating fraction homogenized, then a 1 ml subsample filtered on quartz filters (1.6 mm, 47 mm).	[1]
39 cities, China	Atmospheric fallout (Dust)	Passive Sampling: Hog bristle brushes and pre-cleaned, sealed paper bag with aluminum foil lining for dust collection and storage, respectively, from 39 cities’ bedroom and living room (4 m^2^ each).	Dust sample weighed and placed in a beaker, and 50 mL of ZnCl_2_ solution added for MP density separation. Upper fraction was separated into another tube with a steel spoon and homogenized in 20 mL of the ZnCl_2_ solution. After oscillating, 100 μL aliquots of the solution were added to a grid counter and counted under a light microscope at 100× magnification. Process repeated twice to calculate MP per unit mass of dust.	[14]
Aarhus, Denmark	Aerosol	Active Sampling: Breathing thermal manikin made of aluminum and glass fiber and simulating human presence, and connected to mechanical artificial lung system, consisting of two pneumatic cylinders moved by an electric motor, producing an airflow to simulate breathing with respiration volume of 0.82 L min^−1^.	Filter sonicated for 5 min in pre-cleaned small beaker filled with just enough ethanol (99.9%, HPLC grade) to cover the filter. Membrane then flushed using additional ethanol, after which all the liquid containing the sample was deposited on a pre-heated (55 °C) zinc selenide (ZnSe) window held in a compression cell (PIKE technologies, Fitchburg, WI, USA) using a capillary glass pipette. Enriched ZnSe window dried at 55 °C for 48 h for final sample deposition for determination.	[23]
12 countries; China, Colombia, Greece, India, Japan, Kuwait, Pakistan, Romania, Saudi Arabia, South Korea, USA, and Vietnam	House Dust	Passive Sampling: Nylon brush (China and India) or vacuum cleaner (other 10 countries); indoor pooled floor dust samples were collected from bedrooms and living rooms.	All samples sieved through a 150 μm sieve, and ones <150 µm were collected, homogenized, and stored at 4 °C until analysis. Dust samples (50 mg; spiked with 500 ng D4-TPA and 200 ng 13C12-BPA) weighed, placed in 100 mL round-bottom flask, then both 0.1 g of KOH and 20 mL of 1-pentanol added. The mixture was digested by stirring in heating mantle at 135 °C for 30 min, then allowed to cool down at room temperature while pentanol solution was transferred into a 50 mL PP tube. Flask rinsed twice with 10 mL of HPLC-grade water and rinsate transferred into PP tube. Depolymerized products of PET/PC-based MPs extracted from pentanol by shaking PP tube at 180 strokes per minute for 5 min in orbital shaker (Eberbach Corp., Ann Arbor, MI, USA), followed by centrifugation at 1620× *g* for 5 min (Eppendorf Centrifuge 5804× *g*, Hamburg, Germany). Upper organic phase of pentanol transferred to another tube with 20 mL of HPLC-grade water added and extraction repeated. Aqueous layer (water solution) containing TPA and BPA combined to total volume of 50 mL with HPLC-grade water. A 10 mL aliquot of the solution purified by passing through a SPE cartridge. Dust samples analyzed separately to determine concentrations of freely available TPA and BPA. Briefly, 50 mg of dust sample weighed and transferred into 15 mL PP conical tube. After spiking with 250 ng of D4-TPA and 50 ng of ^13^C_12_-BPA, samples extracted with 5 mL of methanol by shaking in orbital shaker for 30 min. Mixture centrifuged at 2880× *g* for 5 min, and supernatant transferred into new PP tube. Extraction repeated twice with 5 mL of methanol, and extracts combined and concentrated to approximately 1 mL under gentle nitrogen stream. Solution diluted to 5 mL with solvent mixture of methanol and HPLC-grade water at 2:8 ratio (*v*/*v*). Finally, 1 mL diluted solution centrifuged at 9030× *g* for 5 min, then transferred into amber glass vial for HPLC-MS/MS analysis.	[24]
East China Normal University, Shanghai, China	Dust Fallout	Passive Sampling: Fallout into a stainless steel sink; pooled samples collected over 24 h. Samples were collected from dormitory space (25 m^2^, occupied by 2 people), office space (40 m^2^, occupied by 12 people), and spot samples collected in corridor.	Samples collected on filters, carefully removed, and quickly transferred to marked, clean-air sampling cassettes using stainless steel tweezers. Filters examined with stereomicroscope for suspected microplastics and photographed.	[25]
Sydney, Australia	House Dust	Passive Sampling: Fallout was collected into 12 cm glass Petri dishes, placed at 1.2 m height for 30 days. A total of 32 sampling locations in 22 local government areas of metropolitan Sydney.	Sample collected in pre-cleaned, pre weighed Petri dish. Post-collection, the Petri dish was weighed again. The samples were washed from the Petri dish using Milli Q water and filtered under vacuum in a laminar flow unit on a 90 mm diameter glass fiber filter paper of 0.6 µm pore size. Filter paper was marked with 1 cm^2^ grids for ease of navigation under microscope. No sample processing step, i.e., digestion and density separation, was applied.	[26]
Wenzhou, China	Indoor Aerosol	Active Sampling: Sample was collected on 90 mm glass fiber filter with 0.7 µm filter using a LB-120F sampler with flow rate of 100 L min^−1^, a combined 1 m^3^ sample was collected from 39 indoor locations.	Samples collected on 90 mm glass fiber filter with 0.7 µm pore size. Sample was digested in glass beaker using approximately 30 mL 30% H_2_O_2_, heated at 70 °C for 1 h to remove organic matter. The sample was re-filtered on 47 mm diameter PTFE filter membrane with 0.45 µm pore size for Nile red staining. Three drops of 5 mg mL^−1^ Nile red were used for 30 min at room temperature. Samples were digitally photographed using florescence stereo microscope.	[27]
Aveiro, Portugal	Indoor aerosol	Active Sampling: Sample collected using PM10 collector @ 5 L min^−1^ for 48 h on glass fiber filter of 2.2 µm pore size. Sample was collected from from the living room of a two-story house with five residents.	Samples were collected on quartz filter with 2.2 µm pore size at rate of 5 L min^−1^ for 48 h (14.4 m^3^). Sample washed with 15 mL ultrapure water, to which 15 mL of 30% H_2_O_2_ was added and left at room temperature for 8 days to remove organic matter. This digested sample was filtered on glass fiber filter with 1.2 µm pore size, this filter was washed with 1.6 g cm^−3^ NaI solution for density separation. Solution was shaken in vortex for 1 min and left to settle for 90 min, followed by filtration of the supernatant and washing with ultrapure water in glass fiber filters and observed under stereo microscope.	[28]

**Table 4:** Microplastic concentrations and characteristics in indoor aerosols from different locations.

Country	Sample Matrix	MP Concentrations	Size	Shape	Polymer	Color	Reference
Kuwait	Indoor aerosol	3.24 to 27.13 MP m^−3^	0.45–2800 µm	Fibers (91%), fragments (9%)	Polyester, nylon, polyamide	Black, transparent, blue, red, grey	This Study
Paris, France	Indoor aerosol	0.4 to 59.4 fibers m^−3^ Avg. 5.4 fibers m^−3^	50–3250 μm	Fibers	PA, PP	N/A	[1]
39 major cities in China	Fallout (indoor)	PET: 1550–120,000 mg kg^−1^ (average 26,800 mg kg^−1^), geomean (GM) conc. 23,000 mg kg^−1^; PC: (74.4%) <LOQ-107 mg kg^−1^ (average 4.6 mg kg−1), GM 1.8 mg kg^−1^.	50 μm–2 mm	Fibres (88%): 17–620 fibers/mg (Average 342 fibers/mg); granules: 6–184 particles/mg	Polyester, polyacrylonitrile, nylon, polyethylene, polypropylene, poly(ethylene:propylene), acrylic, polyurethane, polyethylenimine, alkyd	N/A	[76]
Aarhus, Denmark	Indoor air	1.7−16.2 particles m^−3^ (average 9.3 ± 5.8 N_MP_ m^−3)^	4−398 µm (Average 177 µm)	Fragments (87%), fibers (13%)	Polyester (81%), polyethylene (6%), nylon (5%), polypropylene (2%), other polymers (6%)	N/A	[23]
Sydney, Australia	Fallout (indoor)	22–6169 fibers m^−2^ d^−1^ (Avg. 3095 fibers m^−2^ d^−1^)	50–2000 µm	7401 fibers, 64 fragments, 18 films.	Fibers were predominantly polyethylene (25%), polyester and PET (17%), polyamide (16%), polyvinyl (15%)	Black, green, blue, red, grey, brown, and transparent.	[26]
Wenzhou, China	Indoor air	1583 ± 1181 MP m^−3^	5–30 µm (60.4 ± 2.7%) 30–100 µm (28.5 ± 2.3%) >100 µm (11%)	Fragments 89.6%, fibers 10.4%	Polyester (28.4%), polyamide (20.54%), polyethylene (16.3%), polystyrene.	NA	[27]
Aveiro, Portugal	Indoor air	6 fibers m^−3^ and smaller 5 MP m^−3^ (6% fibers were synthetic)	17–3669	Fibers	NA	Light color	[28]
12 Countries	Indoor dust	PET—concentrations ranged between 29–120,000 µg/g. Concentrations are arranged in decreasing order of concentration as: South Korea (25,000 µg/g), Japan (23,000 µg/g), Saudi Arabia (13,000 µg/g) Greece (9700 µg/g), Romania (9100 µg/g), United States (8900 µg/g), Kuwait (8600 µg/g), Vietnam (3900 µg/g), China (3700 µg/g) (one sample had 120,000 µg/g, which was 12% of the total mass of dust), Pakistan (1900 µg/g), India (1600 µg/g), Colombia (1000 µg/g). Free TPA median concentrations in dust samples ranged from 2.0 μg/g (Pakistan) to 34 μg/g (Japan). The highest TPA concentration was found in the sample from India (200 μg/g). PC concentration range of <0.11–1700 μg/g. Saudi Arabia (2.5–190 μg/g, median: 45 μg/g) and South Korea (6.7–140 μg/g, median: 38 μg/g) contained the highest concentrations of PC. Free BPA concentrations ranged from <0.05–36 μg/g.	NA	NA	PET, PC		[24]

## Data Availability

Information is available in the manuscript.
